# Phosphorylation of *Beet black scorch virus* coat protein by PKA is required for assembly and stability of virus particles

**DOI:** 10.1038/srep11585

**Published:** 2015-06-25

**Authors:** Xiaofei Zhao, Xiaoling Wang, Kai Dong, Yongliang Zhang, Yue Hu, Xin Zhang, Yanmei Chen, Xianbing Wang, Chenggui Han, Jialin Yu, Dawei Li

**Affiliations:** 1 State Key Laboratory of Agro-Biotechnology and Ministry of Agriculture Key Laboratory of Soil Microbiology, College of Biological Sciences, China Agricultural University, Beijing 100193, China; 2 State Key Laboratory of Plant Physiology and Biochemistry, College of Biological Sciences, China Agricultural University, Beijing 100193, China

## Abstract

Plant virus coat proteins (CPs) play a fundamental role in protection of genomic RNAs, virion assembly, and viral movement. Although phosphorylation of several CPs during virus infection have been reported, little information is available about CP phosphorylation of the spherical RNA plant viruses. Here, we demonstrate that the CP of *Beet black scorch virus* (BBSV), a member of the genus *Necrovirus*, can be phosphorylated at threonine-41 (T41) by cAMP-dependent protein kinase (PKA)-like kinase *in vivo* and *in vitro*. Mutant viruses containing a T41A non-phosphorylatable alanine substitution, and a T41E glutamic acid substitution to mimic threonine phosphorylation were able to replicate but were unable to move systemically in *Nicotiana benthamiana*. Interestingly, the T41A and T41E mutants generated unstable 17 nm virus-like particles that failed to package viral genomic (g) RNA, compared with wild-type BBSV with 30 nm virions during viral infection in *N. benthamiana*. Further analyses showed that the T41 mutations had little effect on the gRNA-binding activity of the CP. Therefore, we propose a model whereby CP phosphorylation plays an essential role in long-distance movement of BBSV that involves formation of stable virions.

*Beet black scorch virus* (BBSV) belongs to the genus *Necrovirus* in the family *Tombusviridae*, and was first reported in the late 1980s in China^1^, followed by identification in the US, Iran and Spain^2^,^3^,^4^,^5^,^6^. Under natural conditions, BBSV-infected beets have scorched and inwardly-curled leaves, and necrotic roots, which result in severe reductions in sugar production^7^. BBSV transmission in the soil is facilitated by zoospores of *Olpidium brassicae*^8^ and at least 15 plants from 4 families, including *Chenopodium amaranticolor*, *Tetragonia expanse*, and *Nicotiana benthamiana*, can be mechanically infected by BBSV^7^,^9^,^10^.

BBSV is a small, approximately 30 nm icosahedral virus, consisting of a 3644 nucleotide (nt) plus-sense, single-stranded (ss) genomic (g) RNA lacking a 5’-cap structure and a 3’-poly(A) tail that encodes six functional proteins. The gRNA serves as the mRNA for the 5’ proximal ORF, which encodes a 23 kDa protein (p23), and an 82 kDa readthrough protein (p82). P23 and p82 interact to form the replicase complex, which is required for gRNA replication and synthesis of two subgenomic RNAs (sgRNAs). Each of the three proteins (p7a, p7b and p5’) translated from sgRNA1 are required for viral cell-to-cell movement and the 24.5 kDa coat protein (CP), which is translated from sgRNA2, is required for systemic infection of *N. benthamiana*, but is dispensable for cell-to-cell movement in *C. amaranticolor*^11^,^12^,^13^.

The CPs of the *Tombusviridae* are composed of an RNA binding domain (R-domain), a shell domain (S-domain), a hinge domain (H-domain) and a protruding domain (P-domain)^14^,^15^. Tombusvirus CPs are also multifunctional and have many important roles during the infection cycle, including nucleic acid binding and encapsidation^16^,^17^,^18^,^19^. CP binding specificity for the gRNA is critical for initiation of virion assembly and is R-domain dependent^20^,^21^. In the case of BBSV, recent studies have demonstrated that an N-terminal region enriched in basic amino acids (^4^KRNKGGKKSR^13^) is involved in CP nuclear localization and gRNA binding, and that lysine and arginine at positions 4 and 5 are key amino acid requirements for gRNA binding^16^,^22^. Although not critical for direct interactions with gRNA, other residues are also required for virion assembly^16^. Mutations of these residues impact virion assembly and viral systemic movement to different degrees, and disruption of these functions supports a model positing that virions are essential for *necrovirus* systemic movement^16^,^23^.

A number of studies have shown that CP stability and functions of diverse plant viruses, such as *Cauliflower mosaic virus* (CaMV), *Potato virus A* (PVA), *Plum pox virus* (PPV), *Bamboo mosaic virus* (BaMV), *Potato virus X* (PVX), *Groundnut bud necrosis virus* (GBNV), and *Brome mosaic virus* (BMV), are regulated by phosphorylation^24^,^25^,^26^,^27^,^28^,^29^,^30^,^31^,^32^. Phosphorylation of the CaMV precapsid protein (p57) by casein kinase II (CKII) activates proteolytic processing to form a “mature” N and C-terminal-truncated p37 product that is essential for successful infection. CP phosphorylation also regulates translation of the gRNAs of several filamentous plant RNA viruses. PVX encapsidated gRNA is completely non-translatable *in vitro*, but when the N-terminal CP residues are phosphorylated by protein kinase C (PKC) or by a casein kinase mixture (CKI and CKII), the RNA is converted to a translatable form^26^. Another case involves the PVA and BaMV CP, in which phosphorylation at the RNA binding domain reduces RNA binding affinity. Both Hung *et al.* (2014) and Ivanov *et al.* (2001 and 2003) hypothesize that CP phosphorylation promotes virion or ribonucleoprotein (RNP) disassembly to release gRNA for replication or translation^27^,^28^,^33^. Hence, several lines of evidence with potexviruses and potyviruses suggest that CP phosphorylation is a general process that regulates co-translational disassembly of virions or RNP, and viral cell-to-cell movement^27^,^28^,^33^.

Although several models have suggested that CP phosphorylation modulates plant virus or RNP disassembly after cell entry, regulation of virion assembly by phosphorylation remains elusive. Weiland *et al.* (2007) have reported that BBSV virions can be phosphorylated^3^, however, more detailed analyses have not been described. In the present study, we provide evidence that the BBSV CP is phosphorylated during infection of *N. benthamiana*. We have also conducted comprehensive biochemical approaches, including mass spectrometry and phosphorylation assays that demonstrate that the CP is phosphorylated at threonine 41 (T41) *in vivo* and *in vitro* by PKA-like kinases. Our results also indicate that CP T41 mutants form incompletely-assembled virus-like particles (VLPs) that are unable to package gRNA and are less stable than wild-type (wt) virions, and that these mutants compromised in systemic movement during infection of *N. benthamiana*. In summary, our results reveal a correlation between CP phosphorylation, virion assembly and long-distance movement, which has been largely unexplored for spherical RNA plant viruses.

## Results

### Identification of phosphorylated residues that affect normal CP functions during BBSV infection

**Figure 1:**
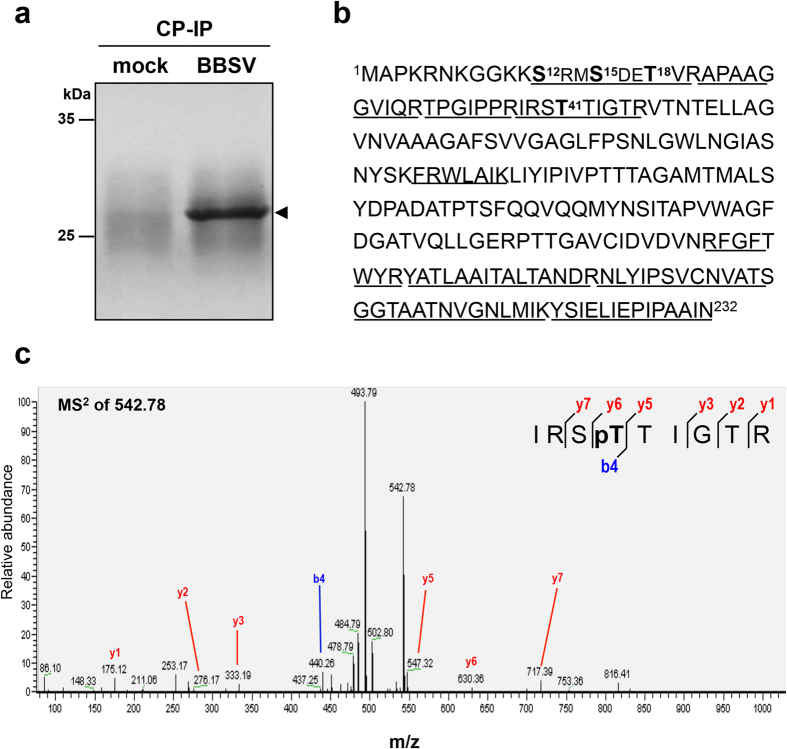
Identification of phosphorylation sites of CP by LC-MS/MS. (**a**) Coomassie blue staining of CP immunoprecipitated (IP) from mock or BBSV inoculated *N. benthamiana* leaf tissues. The CP band (arrowhead) was digested in gel and then subjected to LC-MS/MS analysis. (**b**) Mass spectral sequence coverage (amino acids presented with underline) and identified phosphorylation sites (amino acids presented in bold) in the BBSV CP. (**c**) MS^2^ spectrum of the doubly charged phosphopeptide ^38^IRSpTTIGTR^46^ (m/z 542.78) containing the T41 phosphorylated residue. The colored highlights show b and y ions in the sequence and the corresponding peaks in the spectrum.

**Figure 2:**
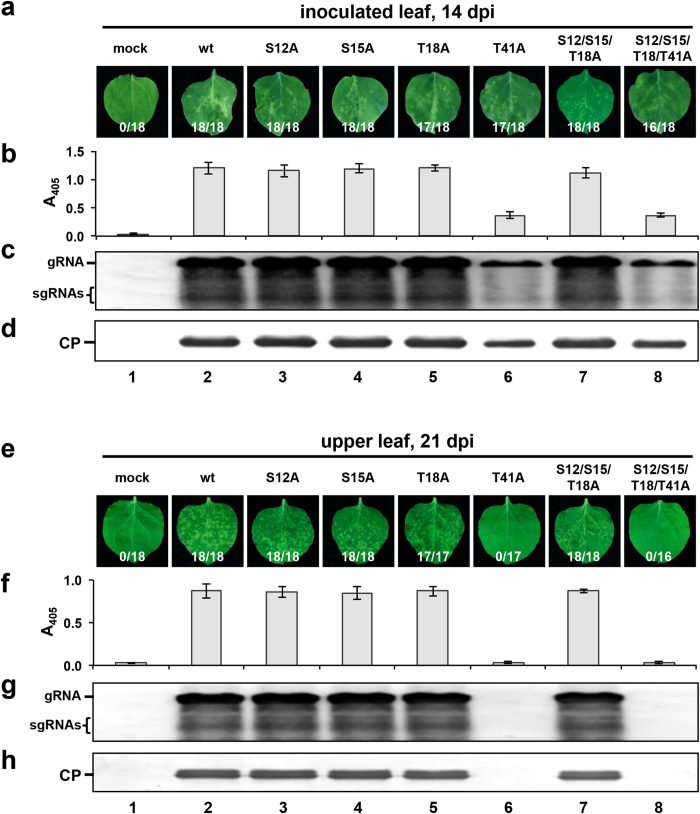
Accumulation of BBSV^wt^ and phosphorylation site mutant viruses in *N. benthanmiana.* *N. benthamiana* leaves exhibiting local (**a**) and systemic (**e**) symptoms after inoculation with BBSV^wt^ and BBSV mutants containing non-phosphorylatable CP substitutions. ELISA detection with CP-specific antibodies of leaf extracts was conducted on inoculated leaves at 14 dpi (**b**) and upper leaves at 21 dpi (**f**). CP titers calculated from A_405_ ELISA values are indicated on the Y-axis. Viral gRNA replication and CP expression were analyzed in inoculated and upper leaves by Northern blot (**c** and **g**) and Western blot (**d** and **h**), respectively. The number ratios of symptom induction among the total inoculated plants are showed at the bottom of panels a and e.

To determine whether the BBSV CP is subjected to phosphorylation during virus infection, CP immunoprecipitated from BBSV-infected *N. benthamiana* leaf tissues was separated by SDS-PAGE (Fig. 1a). After in-gel digestion with trypsin, the CP was analyzed by liquid chromatography–tandem mass spectrometry (LC-MS/MS), and four potential phosphorylation sites, S12, S15, T18, and T41, were identified (Fig. 1b,c). To determine whether CP phosphorylation affects BBSV infection, one or several amino acid residues were substituted with alanines (A), and introduced into the BBSV infectious cDNA clone, pUBF52 (Supplementary Fig. S1). Two-week-old *N. benthamiana* plants were inoculated with BBSV^wt^ or BBSV mutant *in vitro* transcripts, and incubated in a plant growth chamber at 18 °C for about 3 weeks. During early infections, BBSV mutants elicited a local chlorosis on the inoculated leaves similar to that of BBSV^wt^ (Fig. 2a). To detect CP accumulation, leaf tissues were harvested at 14 days post-inoculation (dpi) and subjected to enzyme-linked immunosorbent assays (ELISA, Fig. 2b). CP accumulation of the BBSV^T41A^ (Fig. 2b, lane 6) and BBSV^S12/S15/T18/T41A^ mutants (Fig. 2b, lane 8) was reduced dramatically in comparison to that of BBSV^wt^ (Fig. 2b, lane 2), whereas CP accumulation of BBSV^S12A^, BBSV^S15A^, BBSV^T18A^, and BBSV^S12/S15/T18A^ was similar to that of BBSV^wt^ (Fig. 2b, lanes 3–5 and 7). Viral replication and CP expression were also analyzed by Northern and Western blot, respectively, and consistent results were obtained (Fig. 2c,d). Plants inoculated with the T41A mutants did not developed visible chlorotic spots by 21 dpi, (Fig. 2e, lanes 6 and 8), and ELISA, Northern and Western blot assays did not reveal detectable virus accumulation (Fig. 2f–h, lanes 6 and 8). However, leaves inoculated with other BBSV mutants (Fig. 2e–h, lanes 3–5 and 7) exhibited systemic symptoms and accumulated virus levels similar to those of BBSV^wt^ (Fig. 2e–h, lane 2). These results indicate that phosphorylation of CP T41, but not the other candidate phosphorylation sites, is required for systemic infection of BBSV in *N. benthamiana*.

### CP phosphorylation is associated with PKA-like kinases

**Figure 3:**
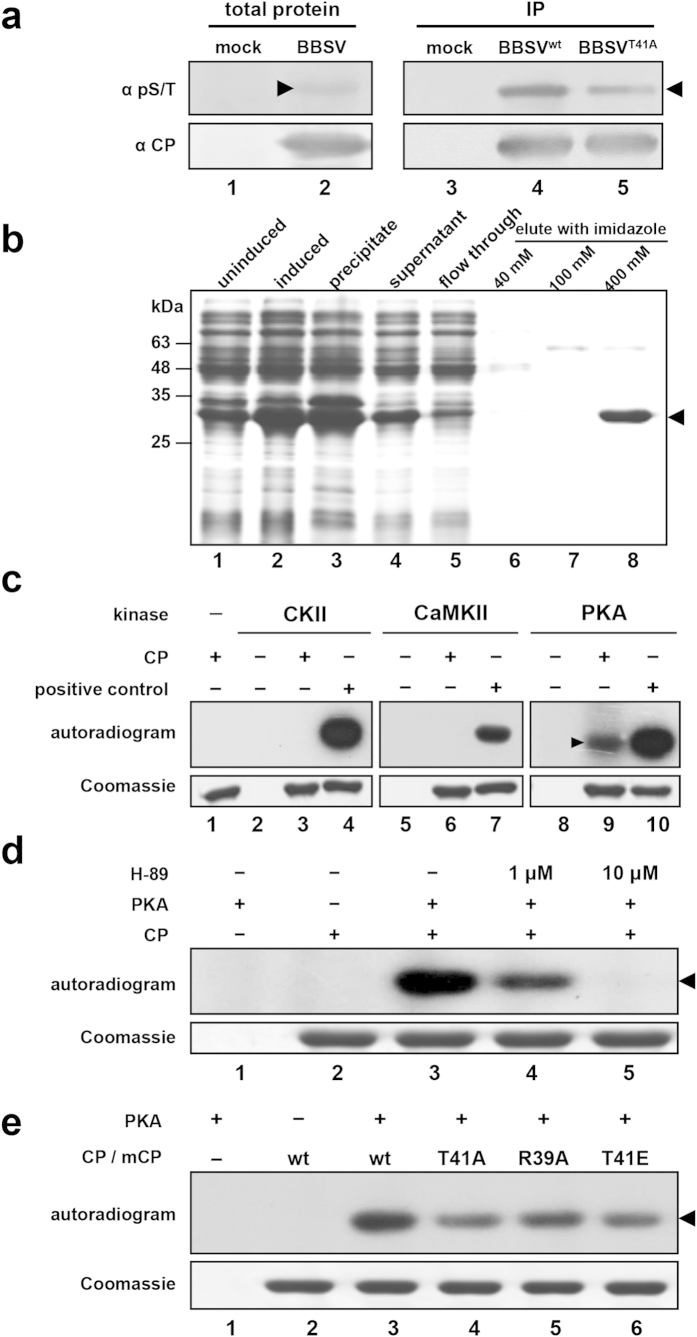
Phosphorylation of BBSV CP by PKA *in vivo* and *in vitro*. (**a**) Detection of phosphorylation of CP by PKA in *N. benthamiana*. Total protein was extracted from BBSV^wt^-inoculated leaves, and subjected to Western blots using phospho-(Ser/Thr) PKA substrate antibodies (α-pS/T, top panel, lane 2) or CP-specific antibodies (α-CP, bottom panel, lane 2). Then total extracted proteins from BBSV^wt^ and BBSV^T41A^ inoculated leaves were also immunoprecipitated with CP-specific antibodies and the same volumes of recovered CP^wt^ and CP^T41A^ were analyzed with CP antibodies (bottom panel, lanes 4 and 5). For the phospho-(Ser/Thr) PKA antibody tests, gels were loaded with three times the amounts used for the CP antibody analyses (top panel, lanes 4 and 5). The mobility of the CP band is indicated by arrowheads, and mock-inoculated *N. benthamiana* extracts served as negative controls (lanes 1 and 3). (**b**) Coomassie blue staining of His-tagged recombined CP (rCP, arrowhead) from uninduced (lane 1) or IPTG induced (lane 2) *E. coli*. The insoluble (lane 3) and soluble (lane 4) rCPs were separated by centrifugation and the soluble fraction was purified by Ni-NTA affinity chromatography (lanes 4–8), and analyzed by SDS-PAGE. Molecular size markers are indicated on the left and the rCP is indicated on the right by an arrowhead. (**c**) *In vitro* phosphorylation of rCP by purified CKII (lane 3), commercial CaMKII (lane 6) and commercial PKA (lane 9, arrowhead). Reactions containing GST-tagged kinase specific substrates served as positive controls (lanes 4, 7, and 10), and reactions lacking rCP (lanes 2, 5, and 8) or specific kinases (lane 1) served as negative controls. The autoradiography (top), and Coomassie blue stained loading controls (bottom) are shown. (**d**) Validation of *in vitro* rCP phosphorylation with the H-89 PKA specific inhibitor. Samples were treated with buffers lacking (lane 3) or containing 1 μM (lane 4) and 10 μM (lane 5) of H-89. Autoradiography and equal loading are shown in the top and bottom panels, respectively. (**e**) *In vitro* phosphorylation assays indicated that T41 is a PKA substrate. His-tagged CP^wt^, CP^T41A^, CP^T41E^ and CP^R39A^ were phosphorylated *in vitro* by PKA and autoradiographed (top panel, arrowhead), and loading controls are indicated at the bottom panel.

According to a previous report^34^ describing phosphorylation specificity of proteins and prediction results from GPS 2.1^35^ (http://gps.biocuckoo.org/index.php) and the online servers KinasePhos^36^ (http://KinasePhos2.mbc.nctu.edu.tw/), the ^38^IRST^41^ region in the N-terminus of BBSV CP is similar to the R-R-X-[S/T] motif, which is a conserved substrate recognition site of cAMP-dependent protein kinase (PKA). To determine whether BBSV CP is phosphorylated by PKA, total protein was extracted from BBSV-infected-*N. benthamiana* leaf tissues at 14 dpi, and subjected to Western blot with a phospho-(Ser/Thr) PKA substrate antibody (α-pS/T) and a CP-specific antibody (α-CP). A weak phosphorylated band corresponding to the CP position was present in the crude extracts from BBSV-infected plants (Fig. 3a, lane 2, top panel), but not from mock-inoculated plant extracts (Fig. 3a, lane 1, top panel). After CP enrichment by CP-specific polyclonal antibody and co-immunoprecipitation with protein G-beads, the phosphorylated CP band displayed higher intensity (Fig. 3a, lane 4, top panel). In addition, phosphorylation of the BBSV CP^T41A^ was approximate 60% of BBSV CP^wt^ immunoprecipitated from inoculated plants (Fig. 3a, lane 5, top panel). These results provided preliminary evidence that the BBSV CP is phosphorylated at T41 by PKA-like kinases in *N. benthamiana*.

To provide additional evidence to confirm that CP is a substrate for PKA, and that T41 is the phosphorylation target site, we performed a series of *in vitro* phosphorylation assays. First, recombinant CP (rCP) fused with an N-terminal hexahistidine tag was expressed in *E. coli* and affinity purified over Ni-NTA agarose (Fig. 3b) and used for *in vitro* phosphorylation assays with commercial PKA (New England Biolabs). As kinase specificity controls, we also used the recombinant *N. benthamiana* CKII (NbCKII) purified as previously described^33^ and a commercial calcium/calmodulin-dependent protein kinase II (CaMKII, New England Biolabs). These experiments revealed that the BBSV CP is specifically phosphorylated by PKA (Fig. 3c, lane 9), but not by NbCKII (Fig. 3c, lane 3) or CaMKII (Fig. 3c, lane 6). To provide additional evidence regarding PKA phosphorylation specificity, the H-89 PKA inhibitor^37^ was added to the reactions (Fig. 3d). In the absence of H-89, an intense radioactive CP band was evident (Fig. 3d, lane 3), but radioactivity was reduced substantially in the presence of 1 μM H-89 (Fig. 3d, lane 4) and was not evident when the H-89 concentration was increased to 10 μM (Fig. 3d, lane 5). Thus the results in total, demonstrate that BBSV CP is a PKA substrate *in vitro*.

In additional experiments, a non-phosphorylatable T41A mutant, a phosphorylation mimicking T41E mutant, and an R39A mutant designed to interfere with the PKA phosphorylation consensus motif R-R-X-[S/T]^34^ were constructed and compared with *in vitro* PKA phosphorylation of rCP^wt^. Three independent experiments consistently revealed that rCP^T41A^, rCP^R39A^, and rCP^T41E^ displayed approximately 50, 67 and 52% phosphorylation efficiency compared with CP^wt^ (Fig. 3e, lanes 3–6). Based on these results, we conclude that CP T41 is phosphorylated *in vivo* and *in vitro* by PKA.

### CP T41 phosphorylation impacts BBSV long-distance movement but not replication

**Figure 4:**
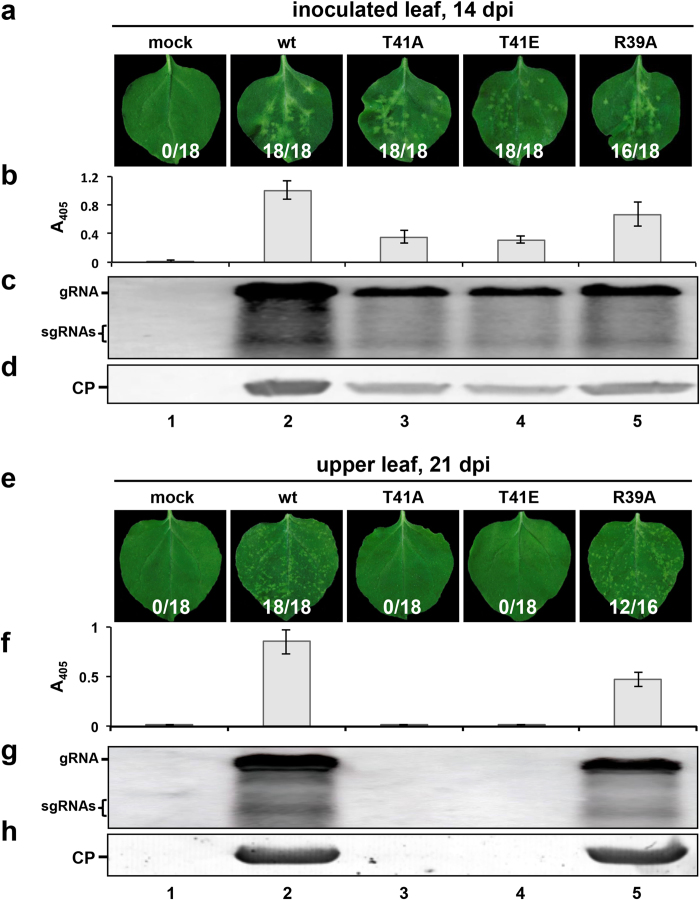
Accumulation of BBSV^wt^ and mutant viruses in *N. benthamiana*. *N. benthamiana* leaves exhibiting local (**a**) and systemic (**e**) BBSV^wt^ and mutant viruses symptoms at 14 and 21 dpi. CP accumulation was determined by ELISA (**b** and **f**) and Western blot (**d** and **h**). Replication of viral RNA in inoculated and upper leaves was examined by Northern blot (**c** and **g**). The infection rate is indicated at the bottom of panels a and e.

To determine whether phosphorylation of CP T41 affects BBSV movement, we compared the symptom induction and accumulation of BBSV^wt^ virions with those of BBSV^T41A^, BBSV^T41E^, and BBSV^R39A^ mutants. Consistent with BBSV^T41A^, which can not be phosphorylated (Fig. 4e–h, lane 3), the BBSV^T41E^ mutant that most closely resembles phosphorylated threonine, did not elicit discernible systemic symptoms (Fig. 4e, lane 4), and did not accumulate detectable CP and viral RNA (Fig. 4f–h, lane 4). These results suggest that interference with T41 phosphorylation impairs systemic infection. Upper leaves of plants inoculated with BBSV^R39A^ developed systemic symptoms, but the proportion of leaves developing symptoms was lower (Fig. 4e, lane 5), and the viral accumulation and infection rates (Fig. 4, lane 5) were also lower than those of BBSV^wt^ (Fig. 4, lane 2). Taken together, these results indicate that phosphorylation of CP at T41 contributes to BBSV long-distance movement in *N. benthamiana*.

**Figure 5:**
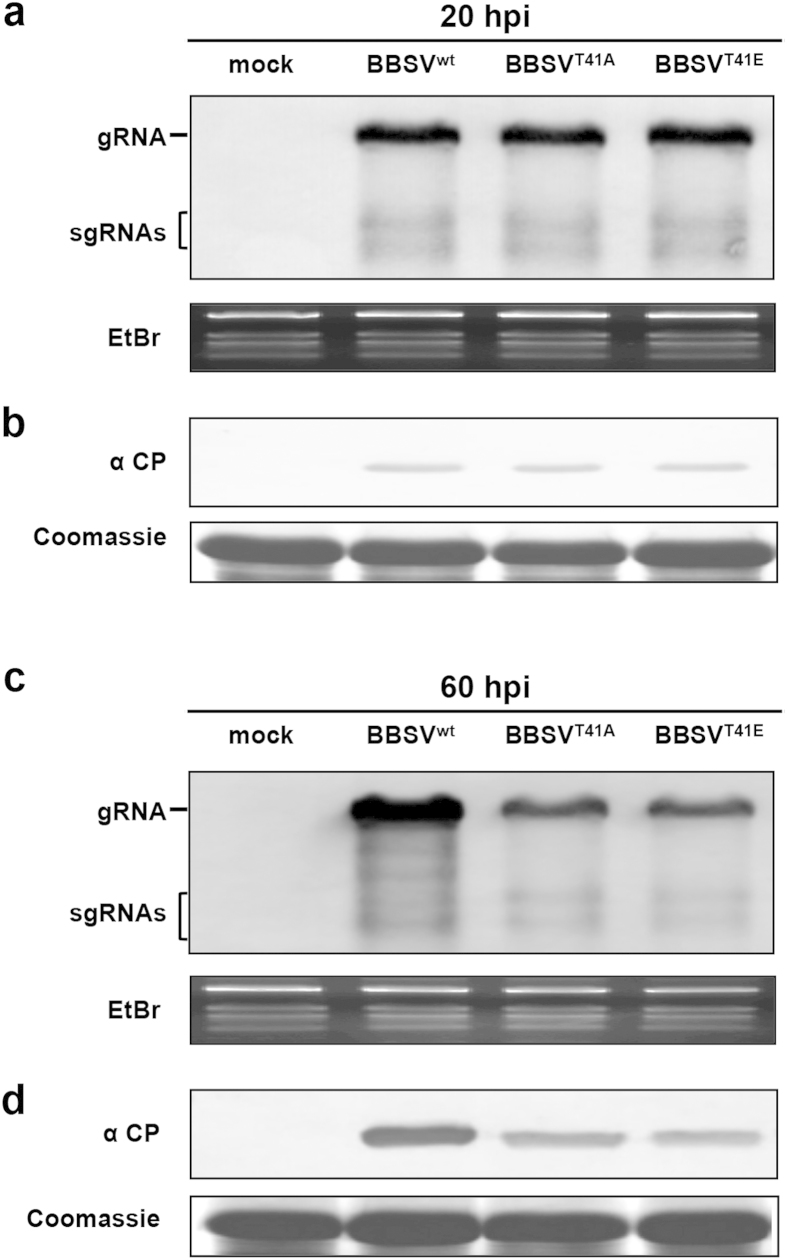
Detection of viral accumulation in *N. benthamiana* protoplasts transfected with BBSV^wt^ and T41 mutant viruses. Total RNA extracted from BBSV^WT^ and T41 mutant inoculated protoplasts at 20 or 60 hpi was subjected to Northern blot with a DIG-labeled BBSV-specific probe (**a** and **c**. top panel). EtBr staining shows the rRNA loading controls (**a** and **c**, bottom panel) and gRNA and sgRNAs are indicated on the left. Total proteins extracted from the protoplasts were subjected to Western blot with CP-specific antibodies (**b** and **d**, top panel). Equal proteins loading is verified by Coomassie blue staining (**b** and **d**, bottom panel), and uninfected protoplasts (mock) served as a negative control.

After determining that CP T41 phosphorylation is involved in BBSV systemic infection, but has little apparent impact on cell-to-cell movement, we were curious about whether CP phosphorylation might have more subtle effects on viral replication. *N. benthamiana* protoplasts were transfected with the BBSV^wt^ or T41 mutants. After 20 hours post-inoculation (hpi), total RNA and protein were extracted from the infected protoplasts and subjected to Northern and Western blot analyses. The accumulation levels of the progeny RNAs (Fig. 5a) and CP (Fig. 5b) of the T41 mutants and BBSV^wt^-infected protoplasts were similar. Hence, phosphorylation of T41 appears not to have obvious effects on the initial accumulation of BBSV RNA or on expression of the BBSV CP. However, by 60 hpi, accumulation of the mutant RNAs was substantially lower than those of BBSV^wt^ (Fig. 5c), and the accumulation levels of the mutant CPs were also lower than the CP^wt^ (Fig. 5d). These results suggest that phosphorylation of T41 does not affect the initial stages of replication, but that viral accumulation is impaired as replication progresses, so it is also possible that CP phosphorylation is required for effective interference with host defense mechanisms at later stages of replication.

### T41 CP mutants are defective in virus assembly

**Figure 6:**
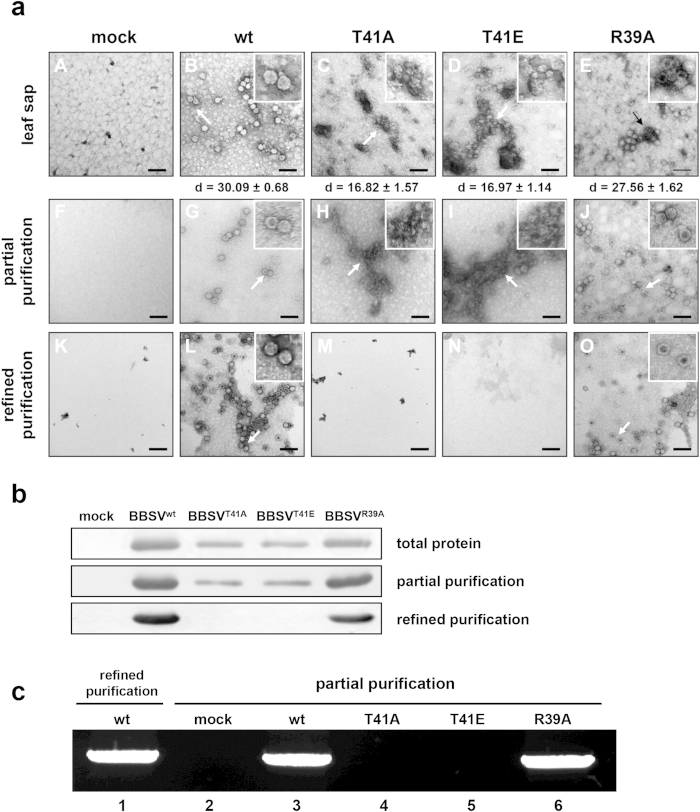
Analysis of particles from *N. benthamiana* leaves inoculated with BBSV^wt^ and mutant viruses. (**a**) Transmission electron microscopy (TEM) of VLPs obtained by partial and refined purification procedures from *N. benthamiana* plants inoculated with BBSV^wt^, BBSV^T41A^, BBSV^T41E^, and BBSV^R39A^ transcripts. Inoculated leaf tissue was harvested at 14 dpi, extracted and observed by TEM (top panel). The VLPs were further enriched by partial or refined purification procedures and detected by TEM (middle and bottom panel). Mock-inoculated leaf samples served as controls (A, F, and K). Bars = 100 nm. More than 50 particles in each sample were measured and their average diameters are shown under the top panel. (**b**) VLP CP detection by Western blot with the CP antibodies used 5 μl samples from partial (middle panel) and refined (bottom panel) purifications. Total protein extracted from inoculated leaf tissues served as controls (top panel). (**c**) EtBr detection of viral RNA from partially purified BBSV^wt^ and mutant preparations (2 μg) after 1% agarose gel electrophoresis (lanes 3–6). BBSV^wt^ virions from the refined purifications served as a positive control to identify the encapsidated RNA (lane 1). Mock-inoculated leaf extracts subjected to the partial purification served as a negative control (lane 2).

It has been reported that stable virions are essential for BBSV systemic movement^16^. Therefore, to determine whether T41 and R39 mutant viruses can form intact virions during infection, BBSV^wt^, BBSV^T41A^, BBSV^T41E^, and BBSV^R39A^-inoculated leaf samples were homogenized in PBS buffer, given a low speed centrifugation, and the clarified supernatants were observed by transmission electron microscopy (TEM) (Fig. 6a, top panel). The mock-inoculated control did not contain visible virus-like particles (Fig. 6a, A), whereas BBSV^wt^ and BBSV^R39A^-infected samples displayed readily observed particles with 30.09 ± 0.68 nm and 27.56 ± 1.62 nm in diameters (Fig 6a, B and E). In contrast, the BBSV^T41A^ and BBSV^T41E^-infected leaf samples contained aberrant particles with diameters of 16.82 ± 1.57 nm and 16.97 ± 1.14 nm, respectively, and these particles tended to form irregular aggregates (n = 50, Fig 6a, C and D). To evaluate the aberrant particles in more detail, partial purifications consisting of a sodium acetate buffer (pH 5) extraction and PEG precipitation were carried out for preliminary enrichment^38^, and the middle panel of Fig. 6a shows that these enriched particles were similar to those in the clarified leaf sap (Fig. 6a, top panel). However, further conventional and refined BBSV^T41A^ and BBSV^T41E^ purifications^39^ did not contain virus-like particles (Fig. 6a, bottom panel, M and N), in contrast to the 30 and 28 nm particles that were readily recovered from the refined BBSV^wt^ and BBSV^R39A^ preparations (Fig. 6a, bottom panel, L and O). We attribute these differences to dissociation of the aberrant particles during extraction in the neutral pH phosphate buffer, which does not contain stabilizing Ca^2+^ ions, and to the subsequent harsh organic solvent clarification. Hence, these results suggest that the BBSV^T41A^ or BBSV^T41E^ mutant virus particles have defects in morphogenesis and are less stable than the BBSV^wt^ particles, and we propose that the systemic movement defects exhibited by T41 mutant viruses are a consequence of incomplete virion formation.

### Aberrant particles formed by T41 mutant viruses are deficient in RNA packaging

To investigate the composition of particles formed by BBSV^wt^ and mutant viruses, particles obtained from the partial and refined purifications were subjected to Western blot analyses using CP-specific antibodies. The results verified that BBSV^wt^ inoculations contained high amounts of CP, whereas the partially purified T41 and R39 mutant BBSV preparations contained lower amounts of CP (Fig. 6b, middle panel). Moreover, the lack of detectable T41 mutant CP in refined purifications strongly suggests that small aberrant virus-like particles are unstable. Interestingly, although BBSV^R39A^ formed virus particles, the particle yield (0.5 mg per 10 g leaf tissue) was less than half the amount recovered from BBSV^wt^ samples (1.35 mg per 10 g leaf tissue) after the refined purification (Fig. 6b, bottom panel), suggesting that the R39 mutation may partially interfere with CP T41 phosphorylation functions *in vivo*, and reduce the accumulation of virus particles.

Because RNA packaging can control the size and shape of virions^21^, we carried out experiments to examine whether the small aberrant T41 particles in the preparations contain viral RNAs, by electrophoresing the same amounts of partially purified particles under non-denaturing conditions, followed by staining of the gels with ethidium bromide (EtBr)^38^,^40^. Only particles formed by BBSV^wt^ and BBSV^R39A^ contained viral gRNAs, as indicated by positive EtBr staining (Fig. 6c, lanes 1, 3, and 6), whereas lanes containing the small virus-like particles formed by the T41A and T41E mutants did not exhibit detectable EtBr staining (Fig. 6c, lanes 4 and 5). These results strongly suggest that the enriched T41A and T41E mutant particles do not contain viral RNA.

**Figure 7:**
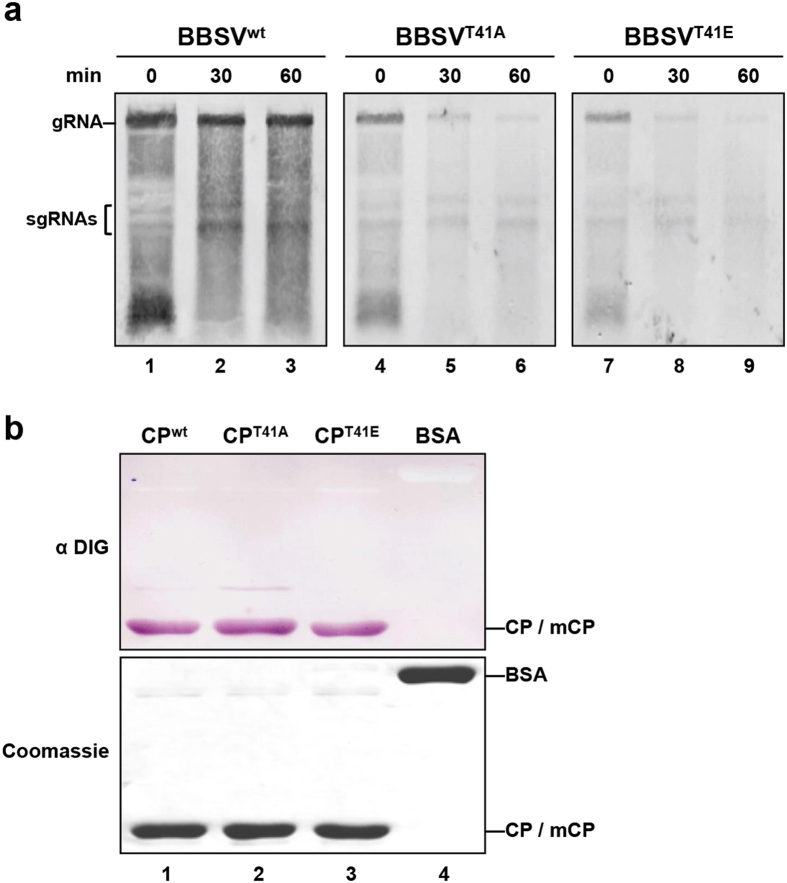
RNA stability in *N. benthamiana* extracts and RNA-binding affinities of wt and T41 mutants. (**a**) RNase protection assay in extracts from *N. benthamiana* infected with BBSV^wt^ and T41 mutants. Inoculated leaves were ground and incubated in PIPE buffer at 37 °C for 30 (lanes 2, 5, and 8) or 60 mins (lanes 3, 6, and 9) to permit endogenous RNase degradation of unprotected RNA. The total RNAs were subsequently extracted and analyzed by Northern blot with a BBSV-specific probe. Bands corresponding to gRNAs and sgRNAs are indicated on the left. Total RNA was extracted as an untreated control immediately after leaves were ground (lanes 1, 4, and 7). (**b**) RNA binding abilities of rCP^wt^ (lane 1), rCP^T41A^ (lane 2), and rCP^T41E^ (lane 3) determined by North-western blot assays with an anti-digoxigenin antibody (α DIG, top panel). BSA served as a negative control (lane 4). The bottom panel shows Coomassie blue stained protein loading controls.

To further evaluate whether viral RNAs are stable after extraction, an RNase-sensitivity assay was carried out by incubating virus-inoculated leaf sap for 30 and 60 min at 37 °C (Fig. 7a). As expected of stable virions, the results showed that, BBSV^wt^ in incubated leaf sap preparations contained the same amounts of viral RNA as extractions made immediately after grinding (Fig. 7a, lanes 1–3). However, after incubation, BBSV^T41A^ (Fig. 7a, lanes 4–6) and BBSV^T41E^ (Fig. 7a, lanes 7–9) gRNAs were degraded by endogenous host RNases present in plant sap. This provides additional evidence that the aberrant virus particles are deficient in morphogenesis steps possibly involved in RNA packaging.

Therefore, we carried out a North-western blot assay^16^ to test whether CP phosphorylation at T41 might affect RNA binding affinity. Compared with the BSA negative control (Fig. 7b, top panel, lane 4), the rCP^wt^ displayed a strong binding affinity for viral RNA (Fig. 7b, top panel, lane l). However, the rCP^wt^, rCP^T41A^, and rCP^T41E^ (Fig. 7b, top panel, lanes 1–3) each had similar RNA-binding affinities. These results thus suggest that phosphorylation of the T41 residue does not directly affect CP RNA binding activity, and that the failure to encapsidate viral RNA occurs at other steps in morphogenesis of the aberrant particles.

## Discussion

Protein phosphorylation, which regulates signal transduction, biological activity, molecular interaction and subcellular localization^34^, is one of the most common posttranslational modifications. Several proteins involved in viral movement are known to be phosphorylated by host kinases, such as the movement proteins (MPs) of the Tobamoviruses, *Tomato mosaic virus* (ToMV) and *Tobacco mosaic virus* (TMV)^41^,^42^,^43^,^44^, and the MPs of *Alfalfa mosaic virus* (AMV), and *Cucumber mosaic virus* (CMV), which belong to the family *Bromoviridae*^45^,^46^. More complex movement proteins are also phosphorylated including the triple gene block 1 (TGB1) protein of *Potato virus X* (PVX), in the family *Alphaflexiviridae*^47^ and TGB1s of *Potato mop top virus* (PMTV) and *Poa semilatent virus* (PSLV) in the family *Virgaviridae*^48^,^49^. Phosphorylation of these MPs by host kinases has been shown to important for virus cell-to-cell movement through regulation of MP stability^42^, cellular localization^42^, RNA binding affinity^48^, and interactions with other MP partners^44^,^49^. These findings suggest that protein phosphorylation has important but poorly understood roles in a number of viral MP processes^50^,^51^.

In addition to the nonstructural MPs, viral coat proteins (CPs) involved in virion or RNP assembly can also affect virus movement through phosphorylation with host serine (S)/threonine (T) kinases. As examples, phosphorylation of CPs of helical PVA and PVX regulates disassembly of RNP or virion to permit replication and translation of viral genomic RNA^26^,^27^,^28^,^33^. However, little is known about the effects of phosphorylation on virion assembly or stability of spherical plant viruses. Our results shown here provide new insight into further understanding of the connection between phosphorylation of the BBSV CP and icosahedral virion assembly.

We found that CP of BBSV is phosphorylated in *N. benthamiana* at four residues, of which only T41 plays a dominant role in viral long-distance movement (Fig. 2). According to the phosphorylation predictions from the KinasePhos online server and GPS 2.1 program, T41 is phosphorylated by PKA. Furthermore, *in vitro* PKA phosphorylation assays indicated that mutations of T41 greatly reduced the CP phosphorylation levels (Fig. 3e). It is plausible that the residual signal is due to phosphorylation of other residues (Fig. 3e, lane 4), and we have found by LC-MS/MS that S15 is also located within a PKA consensus site (Fig. 1b). To further investigate CP phosphorylation, S15A, S12/S15/T18/T41A as well as two negative control mutants, S12A and T18A were constructed. The *in vitro* PKA phosphorylation results with the purified mutant rCPs showed that BBSV CP phosphorylation was reduced significantly in the S15A substitution, compared with that of the rCP^wt^ and the S12A and T18A substitutions (Supplementary Fig. S2, lanes 3, 4, and 6) and incorporation nearly disappeared in the S12/S15/T18/T41A multiple substitution (Supplementary Fig. S2, lanes 5 and 9), Thus, we conclude that S15 residue is also sensitive to PKA. However, the S15 phosphorylation deficient mutant virus has the same local and systemic movement phenotype as BBSV^wt^ suggesting that S15 phosphorylation is not required for long distance movement (Fig. 2, lanes 2 and 4).

Further inoculation analyses suggested that T41 mutations has only minor effects on the replication and translation of viral RNA at 20 hpi of protoplasts, but these mutants had reduced levels of CP and RNA accumulation at 60 hpi (Fig. 5). The T41 mutants also accumulated to lower levels than BBSV^wt^ in inoculated leaves (Fig. 4a–d), and failed to systematically invade upper uninoculated *N. benthamiana* leaves. (Fig. 4e–h).

Examination of particles in leaves inoculated with the BBSV^T41A^ and BBSV^T41E^ mutant viruses revealed that unlike the virions produced by BBSV^wt^, the T41 mutant viruses elicited small irregular spheres that did not contain viral RNA (Fig. 6a,c). Previous studies have shown that RNA folding is a factor that can control particle size and shape^21^,^52^. Thus, the failure to encapsidate gRNA observed in our experiments (Fig. 6c, lanes 4 and 5) could lead to formation of aberrant virus particles. Moreover, RNase-sensitivity assays suggested that gRNAs of the T41A and T41E mutant viruses are susceptible to degradation by endogenous RNases in *N. benthamiana* sap (Fig. 7a). These results indicate that CP T41 mutants are unable to package viral RNAs into intact viral particles or protect against RNA degradation.

We initially hypothesized that the failure to encapsidate RNA might be a consequence of RNA binding defects of the CP mutants. However, the negatively charged mutant (rCP^T41E^) (Fig. 7b, lane 3) displayed similar RNA-binding affinity as that of the non-phosphorylated rCP^wt^ and rCP^T41A^ purified from *E. coli* (Fig. 7b, lanes 1 and 2). This finding is not consistent with previous research on the helical PVA and BaMV virions or RNP in which CP phosphorylation reduces RNA binding activity^27^,^28^,^33^,^53^. However, BBSV virions differ from helical viruses in having an icosahedral *T* = 3 structure in which the major RNA binding domain is located at the basic N-terminal ARM (arginine-rich motif) region^16^. Given that T41 is located 28 amino acids downstream of the major ARM (^4^KRNKGGKKSR^13^) domain^16^, it is not expected to involved directly in RNA binding. Thus, the most logical hypotheses is that phosphorylation of T41 may be indirectly affect RNA binding during packaging, or that phosphorylation may influence dynamic secondary and tertiary precapsid alterations occurring during virus particle morphogenesis.

Several studies have provided evidence that the N-termini of the CPs of some viruses, such as *Sesbania mosaic virus* (SeMV), *Tomato bush stunt virus* (TBSV), CNV, and *Hibiscus chlorotic ringspot virus* (HCRSV) affect the size of the virus particles^54^,^55^,^56^,^57^. For example, the N-terminal ARM and β-annulus are crucial to *T* = 3 virion assembly of CNV, and deletion of these important regions results in the formation of *T* = 1 virus-like particles^58^. Cryo-transmission electron microscopy studies of CNV *T* = 1 and *T* = 3 virus particles reveal that *T* = 3 intact virion is composed of two types of CP dimers which are C-C and A-B subunit dimers. However, *T* = 1 virus-like particle only contains a single subunit dimer that is very similar to the A-B subunit dimers found in *T* = 3 particles. Further analyses demonstrate that the S domain of the C-C dimer have a flat conformation with an ordered N-terminal ARM and β-annulus at the internal surface of particle for RNA packaging, whereas the S domain of A-B dimer is more angled and provides limited space at the N-terminus, which tends to be disordered and limits RNA binding. Although RNA binding plays an important role in regulating the A-B and C-C dimers conformations of CNV, TBSV, and hepatitis E virus (HEV) particles^52^,^56^,^58^, interactions with host factors such as kinases may be involved in stable *T* = 3 particle assembly. Support for this hypothesis comes from several recent findings with hepatitis B virus (HBV)^59^,^60^,^61^. PKA phosphorylation at the N-terminus of HBV core protein accelerates capsid assembly, and is thought to elicit changes in core protein conformation during assembly^59^. The HBV core protein is also phosphorylated by protein kinase C (PKC), which affects core protein subunit affinities, capsid assembly and stability^60^. Thus, phosphorylation is predicted to alter and stabilize the HBV core protein structure during secondary and tertiary interactions that enhance stability of nascent virions^59^,^60^,^61^.

With BBSV, we have carried out an analysis of the crystal structure of an unphosphorylated *T* = 1 virus-like particle assembled from rCP purified from *E. coli* (unpublished data). Consistent with the CP of another necrovirus TNV^15^, the BBSV CP contains an N-terminal R domain, an H domain and a C-terminal S domain. Additionally, like most of *T* = 1 virus particles in *Tombusviridae*^52^,^56^,^58^, we have determined that the N-terminal regions of CP subunits in the BBSV *T* = 1 particle are flexible and disordered, so can not be visualized. However, the T41 residue is visible and is located at a loop region in the H domain that connects the S domain and the β-annulus. We speculate that in the host environment, phosphorylation at T41 might provide a molecular switch that participates in conformation changes of the N-terminus from a disordered state (A-B dimer) to an ordered state (C-C dimer), and that these changes facilitate RNA binding by the N-termini of the C-C dimers and permit final *T* = 3 virion morphogenesis via integration with A-B dimers. It is easy to image that if all of the dimers have the C-C conformation, the redundant ordered N-termini would induce collision in the nascent particles and might interfere with RNA-binding and assembly, as well as steps involved *T* = 3 virion morphogenesis. This hypothesis is supported by the results that both the T41A and T41E mutants formed incompletely-assembled particles that resemble *T* = 1 virions in inoculated leaves and these enriched particles did not contain gRNA. Hence, it is tempting to speculate that dynamic phosphorylation and de-phosphorylation events are required for morphogenesis of *T* = 3 virions.

We have developed an *in vitro* particle assembly assay in preliminary attempts to verify our hypotheses^40^. Unfortunately, in the initial experiments, both PKA phosphorylated rCP^wt^ (Supplementary Fig. S3, C) and non-phosphorylated rCP^wt^ (Supplementary Fig. S3, B) assembled into aberrant virus-like particles with a diameter of 16.5–16.7 nm in the presence of BBSV gRNA. Therefore, in subsequent experiments, we are planning to test viral assembly intermediates that occur during different *in vitro* environments to provide more rigorous analyses of our models for host factor interactions and specific chemical environments that may be required for intracellular virion assembly. Taken together, our current hypotheses are that the formation and stability of intact *T* = 3 particles of BBSV are likely dependent on the complex interactions at the N-terminal that may involve host factors and phosphorylation/de-phosphorylation cycles at T41 to provide molecular switches for virion assembly.

In summary, our study is unique in that it provides the first clues suggesting that phosphorylation of the BBSV CP by a host PKA-like kinase has a critical role in viral morphogenesis, which has not been previously reported in the family *Tombusviridae*. Our data support the notion that the N terminus has important roles in particle assembly and stability. We also hypothesize that the aberrant particles isolated from plants infected with T41 mutants represent *T* = 1 assembly structures without viral RNA that were blocked at an intermediate stage of morphogenesis. These incompletely assembled and unstable particles do not support long-distance movement of BBSV and this provides another avenue for future research.

## Methods

### Immunoprecipitation (IP) and detection of CP phosphorylation

Two grams of inoculated *N. benthamiana* leaves infected with BBSV^wt^ were ground in liquid nitrogen and the resulted powder was dissolved in two volume (w/v) of IP buffer [50 mM Tris-HCl, pH 7.5, 150 mM NaCl, 1% NP-40, EDTA-free protease inhibitor cocktail (Sigma), phosphatase inhibitor (Roche), 10% glycerol]. The homogenate was centrifuged at 4 °C for 30 min at 10,000 *g*, and the supernatant was subjected to two additional centrifugation steps. The final supernatant was incubated with CP-specific antibody (1:2000) at 4 °C overnight, and further enriched by addition of protein G agarose (Millipore) at 4 °C for 4 hours. The immunoprecipiated CP was used for liquid chromatography-tandem mass spectrometry (LC-MS/MS) and immunoblot analyses.

### Identification of CP phosphorylation by LC-MS/MS and immunoblot analyses

For LC-MS/MS identification, immunoprecipitated CP extracted from BBSV inoculated leaf tissues was separated by 12.5% SDS-PAGE and stained with Coomassie brilliant blue R250 (Sigma). After in-gel trypsin digestion and enrichment, the phospho-peptides were separated by nanoscale C18 reverse phase liquid chromatography (Waters), and then electro-sprayed into a Q-Exactive mass spectrometer (Thermo) at the Mass Spectrometry Facility of China Agricultural University. For immunoblot analyses, immunoprecipitated CP was separated by SDS-PAGE and transferred to nitrocellulose membranes (Amersham). A phospho-(Ser/Thr) PKA substrate antibody (Cell Signaling Technology) was used to detect *in vivo* phosphorylation of CP.

### Constructions of plasmids

Several infectious clones used in this study were derived from the infectious cDNA clone of BBSV Ningxia isolate (pUBF52)^11^. To generate phosphorylation mutants, Quick-change site-directed mutagenesis was used with self-complementary primers (Supplementary Table S1). For expression of CP and its mutants for *in vitro* phosphorylation assays, wt and mutant fragments were amplified with appropriate primers listed in Supplementary Table S1 and cloned into the pET-30a (+) vector (Novagen). To prepare specific substrates for selected kinases, the oligos corresponding to peptides “RRADDSDDDDD” for CKII, “LRRASLG” for PKA and “KKALRRQETVDAL” for CaMKII were inserted into the pGEX-KG vector^62^ with an N-terminal glutathione S-transferase (GST) tag using reverse PCR primers (Supplementary Table S1).

### Virus inoculation and molecular analysis of progeny viruses

Mechanical inoculation of various mutants onto 2-week-old *N. benthamiana* plants was performed according to a previous study^11^, except that all the *in vitro* transcripts were adjusted to equal amounts by UV spectrophotometry to ensure uniformity of the experiments. At 12 and 24 dpi, total RNA and protein was extracted from infected leaves and prepared for further analyses.

For protoplast transfections, mesophyll protoplasts were prepared from the second and third true leaves of 3 to 4-week-old *N. benthamiana*^63^. Approximately 10^6^ isolated protoplasts were transfected with 20 μg of purified transcripts according to a PEG-calcium-mediated transfection method^39^. After culturing for 20 or 60 hours, total RNA was extracted from the transfected protoplasts with Trizol reagent (Invitrogen) and prepared for further analyses.

Northern blots were conducted according to the manufacturer instructions (DIG high prime DNA labeling and detection kit I, Roche) and DIG-labeled probe was prepared from a DNA fragment corresponding to the 3¢ UTR of the BBSV genome^12^. Western blots and (enzyme-linked immunosorbent assays) ELISA were performed using CP-specific antibodies as described previously^64^.

### Expression and purification of recombinant protein

*E. coli* strain BL21 (DE3) pLysS cells (Novagen) were transformed with pET30a-CP^wt^ or phosphorylation mutant constructs by heat shock, and cultured at 37 °C until the OD_600_ reached 0.4. Then, 0.2 mM IPTG (Sigma) was used to induce protein over-expression at 18 °C for another 18 hours. Bacteria cells were then harvested, resuspended in T buffer (20 mM Tris-HCl, pH 7.5, 500 mM NaCl, 10% glycerol, 1 mM PMSF) containing 0.1% Triton X-100 and disrupted by ultrasonication. After centrifugation for 20 min at 20,000 *g*, the supernatant was collected to purify the N-terminal hexahistidine tagged CP by Ni-NTA agarose affinity (Bio-Rad). After washing with increasing step-wise concentrations of imidazole, proteins eluting in T buffer containing 400 mM imidazole were concentrated with 30 kDa Amicon-Ultra-15 filters (Millipore).

Three pGEX-KG^60^ based constructs of kinase specific substrates (subCKII, subPKA and subCaMKII) were transformed into *E. coli* strain BL21 (DE3) pLysS cells. After culturing and induction, proteins were purified from the cells by affinity chromatography on glutathione affinity sepharose columns (GE healthcare). GST-tagged proteins were eluted by T buffer containing 2 mM DTT and 60 mM L-Glutathione and concentrated with Amicon-Ultra-15 filters (30 kDa). Purified protein concentrations were determined by measuring the OD_280_ with a NanoDrop ND1000 spectrophotometer, and proteins were assessed by SDS-PAGE.

### *In vitro* phosphorylation assays

Five μg of rCP or kinase specific substrates and 0.5 μg of kinase [purified NbCKII or commercial mammal kinases PKA or CaMKII (New England Biolabs)], were incubated in 10 μl reactions containing 1X kinase reaction buffer (New England Biolabs), 3 μCi γ-^32^P-ATP (Perkin Elmer) and 200 μM unlabeled ATP. To inhibit PKA phosphorylation, 1 μM of PKA specific inhibitor H-89 [5-Isoquinolinesulfonamide, N-(2-((3-(4-bromophenyl)-2-propenyl)amino)ethyl)] was added. After incubation at 30 °C for 30 min, the reactions were quenched by addition of 5X SDS sample buffer (250 mM Tris-HCl, pH 6.8, 10% SDS, 50% Glycerol, 0.5% Bromophenol blue, 5% β-mercaptoethanol) and boiled for 5 min. Then samples were separated by 12.5% SDS-PAGE followed by autoradiography.

### Virus particle purification

Three procedures were used to obtain BBSV^wt^ and mutant viral particles for Transmission electron microscopy (TEM), Western blots and agarose gel electrophoresis analyses.

To rapidly obtain viral particles, virus particles in clarified leaf sap were extracted from one gram of *N. benthamiana* leaves inoculated with BBSV^wt^ or mutant viruses at 14 dpi by grinding in 200 μl of 20 mM PBS (pH 7.0) followed by centrifugation at 4 °C for 30 min at 6,000 *g*. The supernatants were collected and used for various analyses described in the Results.

A previously described partial purification procedure was used to concentrate virus particles^38^. Briefly, 0.5 gram of virus-inoculated leaves were collected, ground in liquid nitrogen, resuspended at 4 °C in 2 ml of 0.1 M sodium acetate buffer (pH 5.0) containing 5 mM β-mercaptoethanol, and the mixture was centrifuged at 4 °C for 30 min at 13,000 *g*. Then the supernatant was adjusted to 8% PEG_6000_ and gently shaken for 30 min at 4 °C. The homogenate was centrifuged at 4 °C for 30 min at 13,000 *g*, and the pellet containing viral particles were resuspended in 50 μl ddH_2_O. For subsequent analyses to visualize virus particles, 5 μl of partial purified particles were subjected to Western blots with a CP-specific antibody. To detect viral gRNA, 2 μg particles were separated by 1% agarose gel in electrophoresis buffer (0.1 M sodium acetate, pH 6.0, 1 mM EDTA) and stained with EtBr.

To obtain more entensively purified viral particles, a conventional and refined purification procedure was performed as described earlier^16^. Ten grams of virus-inoculated leaves were collected and ground in a blender with two volumes (w/v) of 0.2 M sodium phosphate buffer (pH 7.0) containing 0.2% β-mercaptoethanol. The mixture was then stirred continuously on ice for one hour, and centrifuged for 30 min at 10,000 *g* to remove the insoluble material. The supernatant was collected, mixed with 15% chloroform, and centrifuged at 4 °C for 30 min at 13,000 *g*. Then the supernatant was collected, mixed with 6% PEG_6000_ and 3% NaCl, stirred for 2 hours and stored overnight at 4 °C. For virus precipitation, the homogenate was centrifuged for 30 min at 13,000 *g* and the pellet was thoroughly resuspended in 0.2 M sodium phosphate buffer (pH 7.0) containing 0.1% TritonX-100. The clarified supernatant was centrifuged at 4 °C for 90 min at 32,000 *g* and the final virion pellet was resuspended in 100 μl ddH_2_O.

### Transmission electron microscopy (TEM)

TEM was carried out according to methods described previously^64^. Briefly, 200 mesh carbon-coated nickel grids were sequentially incubated with BBSV CP-specific antibody and purified particles, followed by staining of the grids with 1% uranylacetate and visualization with a transmission electron microscope (JEM-1230, JEOL Co. Ltd, Japan) operated at 80 kV.

### RNA binding assay (North-western blot assay)

Five μg of purified rCPs or BSA, which served as negative control, were separated by 12.5% SDS-PAGE and transferred to nitrocellulose membrane. The proteins were renatured by incubating the membrane overnight at 4 °C in 20 ml of renaturation buffer (50 mM Tris-HCl, pH 7.5, 0.1%TritonX-100, 10% glycerol, 0.1 mM ZnCl2 and 250 mM KCl). Then, a digoxigenin (DIG)-labeled North-western blot procedure was performed as previously described^16^.

### RNase-sensitivity assays

The stability of virus particles to endogenous RNases in leaf sap was determined by an RNase-sensitivity assay as previously reported^16^,^65^ with minor modifications. Briefly, 0.2 gram of virus-infected leaves were ground in liquid nitrogen and incubated in 200 μl PIPES buffer (50 mM PIPES, pH 6.5, 0.1% Tween20) at 37 °C for 30 or 60 min. Then, total RNA was extracted using Trizol reagent. Untreated samples, whose total RNA was extracted immediately served as controls. The amount of remaining viral RNA was analyzed by Northern blot as described above.

## Additional Information

**How to cite this article**: Zhao, X. *et al.* Phosphorylation of *Beet black scorch virus* coat protein by PKA is required for assembly and stability of virus particles. *Sci. Rep.* **5**, 11585; doi: 10.1038/srep11585 (2015).

## Supplementary Material

Supplementary Information
