# Supplementary material for: Phosphorylation of Beet black scorch virus coat protein by PKA is required for assembly and stability of virus particles
Source: Sci Rep. 2015 Jun 25;5:11585. doi: 10.1038/srep11585 (PMC4479801; doi:10.1038/srep11585)
Supplement: Supplementary Information [file srep11585-s1.pdf]

Phosphorylation of *Beet black scorch virus* coat protein by PKA is required for assembly and stability of virus particles

Xiaofei Zhao[1], Xiaoling Wang[1], Kai Dong[1], Yongliang Zhang[1], Yue Hu[1], Xin Zhang[1], Yanmei Chen[2], Xianbing Wang[1], Chenggui Han[1], Jialin Yu[1] & Dawei Li[1]*

Supplementary information

Supplementary Figures

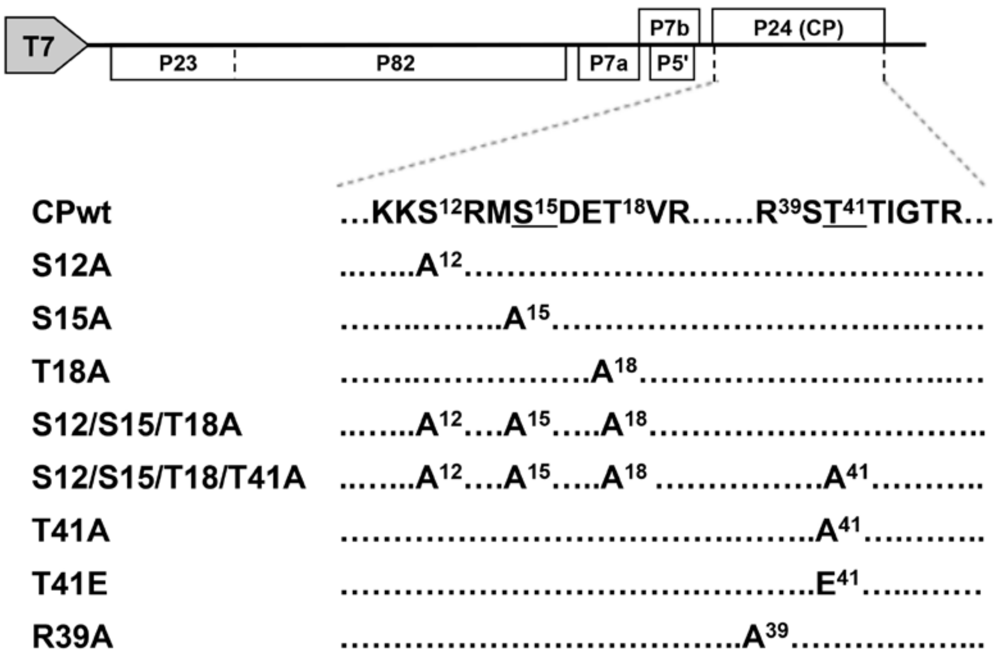

Supplementary Fig. S1 Schematic representation of CP phosphorylation mutations incorporated into the infectious BBSV cDNA clone (pUBF52).

The BBSV gRNAs are *in vitro* transcribed under control of the T7 promoter. The 5' proximal gRNA ORFs encodes p23 and readthrough p82 proteins that interact to form the replicase complex for synthesis of gRNA and sgRNAs. The 3' half of the genome encodes three movement proteins, p7a, p7b, and p5' that are translated from sgRNA1, and p24 (CP) which is translated from sgRNA2. The CP N-terminus (aa 10-20 and aa 39-46) containing the candidate phosphorylation sites and their substitution derivatives are illustrated above.

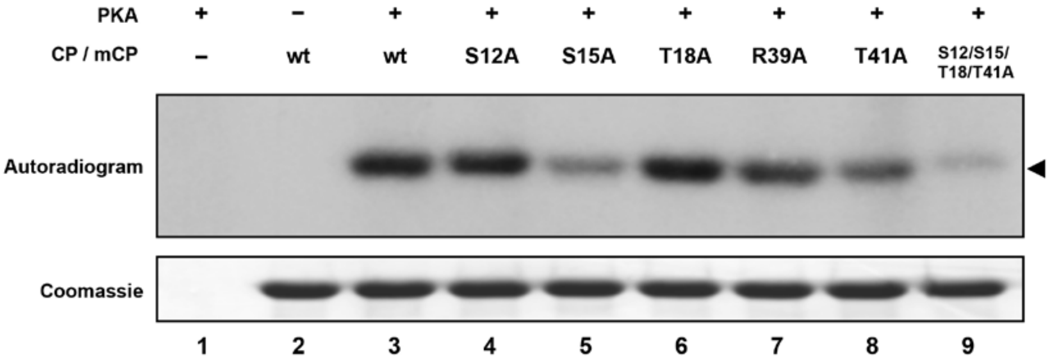

Supplementary Fig. S2 PKA phosphorylation of CP at T41 and S15.

In vitro phosphorylation of rCP^{wt} and CP mutants showing that S15 and T41 are phosphorylated by commercial PKA. His-tagged CP^{wt}, CP^{S12A}, CP^{S15A}, CP^{T18A}, CP^{R39A}, CP^{T41A}, and CP$^{S12/S15/T18/T41A}$ were phosphorylated *in vitro* by PKA and autoradiographed (top panel, lanes 3-9, arrowhead). Reactions lacking rCP (lane 1) or PKA (lane 2) served as negative controls. Equal loading is indicated at the bottom panel.

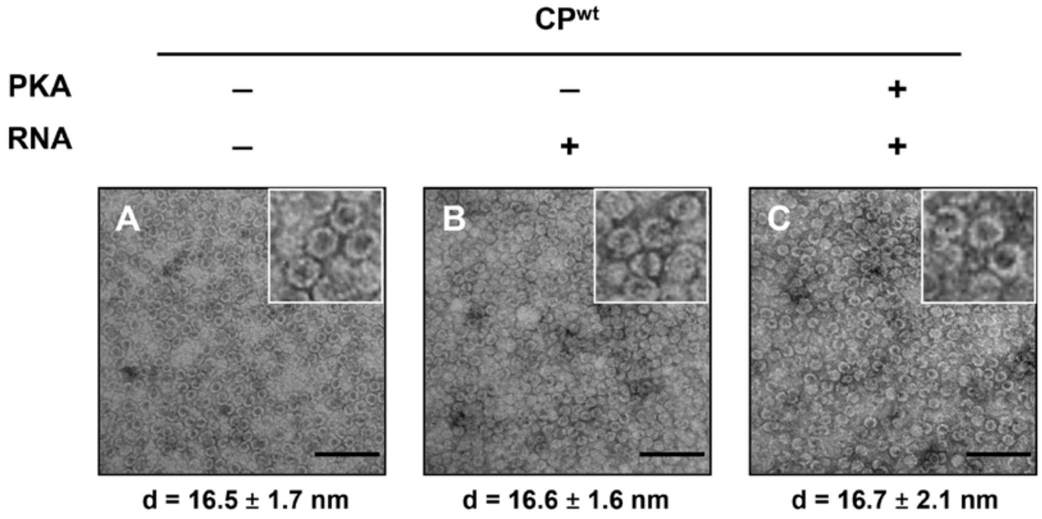

Supplementary Fig. S3 *In vitro* assembly of virus-like particles from rCP^{wt} and BBSV *in vitro* transcripts.

Phosphorylated rCP^{wt} and *in vitro* BBSV transcripts were mixed and subjected to *in vitro* assembly (C) as described in the supplementary methods below. Assembly of non-phosphorylated rCP^{wt} with (B) or without (A) RNA served as control. Subsequently, the samples were concentrated and observed under TEM and the sizes of 50 particles were measured.

Supplementary Methods

In vitro assembly of virus-like particles

Recombinant CP^{wt} (rCP^{wt}) was purified from *E. coli* and subjected to *in vitro* PKA phosphorylation as described in the text of the primary paper. Subsequent assembly reactions used minor modifications of the procedure of Cadena-Nava *et al.*[1]. Briefly, 300 μg of phosphorylated rCP^{wt} was mixed at a ratio of 6:1 (wt/wt) with purified *in vitro* BBSV transcripts (50 μg). The reaction mixtures were then dialyzed overnight at 4°C against assembly buffer (50 mM Tris-HCl, pH 7.2, 10 mM KCl, 5 mM $MgCl_2$, 1 mM DTT). The overnight dialysis was then followed by dialysis for 6 hours at 4°C in reassembly buffer (50 mM sodium acetate, pH 4.8, 5 mM $MgCl_2$, 1 mM DTT). The assembled particles were subjected to five washes in involving resuspension in reassembly buffer and centrifugation (3000 *g*) at 4°C with a Amicon-Ultra-0.5 filter (100 kDa, Millipore)[1].

References

1 Cadena-Nava, R. D. *et al.* Self-assembly of viral capsid protein and RNA molecules of different sizes: requirement for a specific high protein/RNA mass ratio. *J. Virol.* **86**, 3318-3326 (2012).

Table S1. Primers used for construction of mutant BBSV infectious clones and prokaryotic expression vectors.

clone	primer[a]	sequence (5′-3′)[b]	purpose
pUBF52 mutants			
**CP-S12A**	qcS12A-F	AGGAGGCAAGAAGGCCCGCATGTCCGATGAGACAGTG	S12 substituted by A using Quick-change PCR
	qcS12A-R	CACTGTCTCATCGGACATGCGGGCCTTCTTGCCTCCT	
**CP-S15A**	qcS15A-F	AGGAGGCAAGAAGTCCCGCATGGCCGATGAGACAGTG	S15 substituted by A using Quick-change PCR
	qcS15A-R	CACTGTCTCATCGGCCATGCGGGACTTCTTGCCTCCT	
**CP-T18A**	qcS15A-F	CCGCATGTCCGATGAGGCAGTGCGGGCTCCTGC	T18 substituted by A using Quick-change PCR
	qcS15A-R	GCAGGAGCCCGCACTGCCTCATCGGACATGCGG	
**CP-S12/S15/T18A**	qc121518A-F	GGAGGCAAGAAGGCCCGCATGGCCGATGAGGCAGTGCGGGCTCC	S12, S15 and T18 substitued by A using Quick-change PCR
	qc121518A-R	GGAGCCCGCACTGCCTCATCGGCCATGCGGGCCTTCTTGCCTCC	
**CP-R39A**	qcR39A-F	GCATTCCTCCCCGCATTGCGTCCACCACTATTGGTACG	R39 substituted by A using Quick-change PCR
	qcR39A-R	CGTACCAATAGTGGTGGACGCAATGCGGGGAGGAATGC	
**CP-T41A**	qcT41A-F	CGCATTAGGTCCGCCACTATTGGTACG	T41 substituted by A using Quick-change PCR
	qcT41A-R	CGTACCAATAGTGGCGGACCTAATGCG	
**CP-T41E**	qcT41E-F	CCTCCCCGCATTAGGTCCGAGACTATTGGTACGC	T41 substituted by E using Quick-change PCR
	qcT41E-R	GCGTACCAATAGTCTCGGACCTAATGCGGGGAGG	

pET30a-CP mutants			
**CP wt/mutants**	CP-F	CAGTGGATCCATGGCACCTAAGCGCAAT	forward primer contains *Bam*HI
	CP-R	TGACGTCGACCTAATTAATGGCAGCAGGTA	reverse primer contains *Sal*I
pGEX-KG-substrate			
**CKIIsub**	CKIIsub-F	GACGACGACGACGACTAGCTGGTTCCGCGTGGATCCCCG	Insert peptide "RRADDSDDDDD" followed GST-tag using reverse PCR
	CKIIsub-R	ACTATCATCAGCGCGGCG ATCCGATTTTGGAGGATGGTCGCC	
**PKAsub**	PKAsub-F	AGCCTCGGA TAG CTGGTTCCGCGTGGATCCCCG	Insert peptide "LRRASLG" followed GST-tag using reverse PCR
	PKAsub-R	AGCACGTCGC AG ATCCGATTTTGGAGGATGGTCGCC	
**CaMKIIsub**	CaMKIIsub-F	GAGACTGTAGACGCACTGTAGCTGGTTCCGCGTGGATCCCCG	Insert peptide "KKALRRQETVDAL" followed GST-tag using reverse PCR
	CaMKIIsub-R	TTGCCGTCGGAGAGCCTTCTTATCCGATTTTGGAGGATGGTCGCC	

[a] F, forward primer; R, reverse primer.

[b] Restriction sites are underlined.
